# Introduction to the virtual thematic issue on room-temperature biological crystallography^1^

**DOI:** 10.1107/S2052252523002968

**Published:** 2023-03-31

**Authors:** Roberto A. Steiner

**Affiliations:** a King’s College London, New Hunt’s House - Guy’s Campus, London SE1 1UL, United Kingdom; b Department of Biomedical Sciences, University of Padova, via Ugo Bassi 58/B, Padova, 35131, Italy

**Keywords:** room-temperature crystallography, synchrotron radiation, serial crystallography, protein dynamics

## Abstract

Room-temperature biological crystallography has seen a resergence in recent years and a collection of articles recently published in *IUCrJ*, *Acta Cryst. D Structural Biology* and *Acta Cryst. F Structural Biology Communications*, have been collected together to produce a virtual special issue at https://journals.iucr.org/special_issues/2022/RT/.

After many years in which the use of cryogenic conditions (typically around 100 K) represented an almost ubiquitous approach to biological crystallographic studies, there has been a recent resurgence in room temperature (RT) or (near-)physiological temperature experimentation. One of the main advantages of measuring diffraction data at low temperatures is the possibility of mitigating the detrimental effects of radiation-induced damage in macromolecular crystals (Garman & Schneider, 1997 ▸). Although cryo-conditions do not completely abolish global and specific radiation damage (Bui *et al.*, 2014 ▸; Garman, 2010 ▸; Ravelli & McSweeney, 2000 ▸; Weik *et al.*, 2000 ▸), their use, together with judicious instrument choices, often allow the collection of complete diffraction data from a single crystal without significant loss in resolution. Nowadays, whilst the ‘one crystal, one data set’ experimental format afforded by cryo-temperatures remains popular and practically convenient, advances in multi-crystal serial data collection approaches (SFX and SSX for serial femtosecond and serial synchrotron X-ray crystallography, respectively) and detectors with extremely fast read-out rates allow the measurement of complete datasets at RT with modest radiation-induced damage. These developments offer exciting opportunities as there is an increasing realization that RT or multi-temperature measurements, represent a valid approach to study functionally important conformations that may be hidden at cryogenic temperatures (Fraser *et al.*, 2009 ▸; Keedy *et al.*, 2018 ▸; Keedy *et al.*, 2015 ▸; Keedy *et al.*, 2014 ▸; Fenwick *et al.*; 2014 ▸). Also, temperature-dependent differences in protein-ligand interactions including unique binding poses and new binding sites (Skaist Mehlman *et al.*, 2023 ▸) make RT crystallographic studies very relevant for a more complete understanding of protein–ligand interactions that might be important for drug discovery (Fischer *et al.*, 2014 ▸; Fischer *et al.*, 2015 ▸). Importantly, RT studies on microcrystals coupled with suitable mixing and/or activation methods can also allow the visualization of reaction intermediates (Olmos *et al.*, 2018 ▸; Butryn *et al.*, 2021 ▸; Gruhl *et al.*, 2023 ▸).

This virtual thematic issue (see https://journals.iucr.org/special_issues/2022/RT/) presents a collection of articles recently published in *IUCrJ*, *Acta Cryst. D Structural Biology* and *Acta Cryst. F Structural Biology Communications*, some of which have been commissioned specifically, that highlight topics and trends in modern RT biological crystallography.

In a review paper, Thorne provides an overview of the key advantages and physical principles of, and methods for, crystallographic data collection at non-cryogenic temperatures (Thorne, 2023 ▸). The paper also discusses some factors relevant to interpreting the resulting data. This review complements another excellent review on practical methods for RT data collection and analysis published recently in *Quarterly Reviews of Biophysics* (Fischer, 2021 ▸).

A series of original research articles then discuss RT crystallographic work in the context of different biological systems. Metal centres in proteins can be very sensitive to radiation damage thus making standard single crystal RT experiments particularly challenging. Moreno-Chicano and co-workers explored the complementarity of several diffraction techniques to study the multifunctional heme-containing dehaloperoxidase B (DHP-B) globin (Moreno-Chicano *et al.*, 2022 ▸). Aumonier and colleagues used RT time-resolved oscillation crystallography coupled with *in crystallo* UV–Vis absorption spectroscopy to look at the slow relaxation of a reaction intermediate – a thio-ether covalent bond between the chromophore and a cysteine residue – in the photoreaction of the LOV2 domain of phototropin 2 from *Arabidopsis thaliana* (Aumonier *et al.*, 2022 ▸). Ebrahim and co-workers performed a multi-temperature experiment on the SARS-CoV-2 main protease (M^pro^), a promising target for the development of novel antiviral therapeutics (Ebrahim *et al.*, 2022 ▸). Using multi-copy ensemble models they highlight conformational heterogeneity at various sites including a key substrate-binding loop that may inspire new strategies for antiviral drug development. Sharma and co-workers further explored the theme of conformational variability and allosteric coupling in protein tyrosine phosphatase 1B (PTP1B) by solving its SSX structure in the unliganded state (Sharma *et al.*, 2023 ▸) whilst Schneps and colleagues employed SSX on the circadian photoreceptor cryptochrome (CRY) revealing regions of enhanced mobility in loops that have functional importance within this protein family and whose atomic displacement parameters correlate well with those predicted from molecular-dynamics simulations (Schneps *et al.*, 2022 ▸). The theme of ligand-binding discrepancies between cryo-cooled and physiological temperature structures is discussed by Huang and co-workers by looking at the interaction of TL00150, a 175.15 Da fragment, with endothia­pepsin using a ‘temperature-resolved’ approach (Huang *et al.*, 2022 ▸). Some aspects of radiation damage are considered by Yabukarski and colleagues who employed a panel of well known model systems, to show that radiation damage only modestly increases conformational heterogeneity at RT, suggesting that dynamic information can be obtained from single crystals at near-physiological temperature (Yabukarski *et al.*, 2022 ▸). Båth and co-authors employed lipidic cubic phase (LCP) microcrystallization applied to the photosynthetic reaction centre of *Blastochloris viridis* (Båth *et al.*, 2022 ▸). Using X-ray free electron laser (XFEL) radiation they obtained its structure at the 2.25 Å resolution that allowed the visualization of the mobile ubi­quinone potentially facilitating time-resolved diffraction studies of electron-transfer reactions to this co-factor. Finally, Zielinski and co-workers discussed efficient RT SSX experiments carried out on three different proteins of biological relevance using the CFEL TapeDrive, a conveyor-belt-based sample-delivery system (Zielinski *et al.*, 2022 ▸).

The issue also includes a few articles that are a bit more ‘technical’ in their nature. Greisman and colleagues discuss native single anomalous diffraction (SAD) phasing at RT (Greisman *et al.*, 2022 ▸) whilst Doukov and co-workers show how high-quality anomalous signal can be collected from single-crystal diffraction data with relatively low occupancy at and above RT (Doukov *et al.*, 2023 ▸). In the field of synchrotron instrumentation, increasingly more sophisticated developments are happening at sites worldwide. One example is discussed by Okumura and colleagues in their presentation of the setup for *in situ* crystal data-collection and ligand-screening available at SPring-8 (Okumura *et al.*, 2022 ▸).

Hopefully, this virtual thematic issue will encourage the reader to explore additional published work on the topic of RT biological crystallography and, importantly, facilitate the conception and execution of relevant experiments.
